# Myalgic encephalomyelitis/chronic fatigue syndrome (ME/CFS): A preliminary survey among patients in Switzerland

**DOI:** 10.1016/j.heliyon.2023.e15595

**Published:** 2023-04-20

**Authors:** Rea Tschopp, Rahel S. König, Protazy Rejmer, Daniel H. Paris

**Affiliations:** a Swiss Tropical and Public Health Institute, Kreuzstrasse 2, 4123 Allschwil, Switzerland; b Faculty of Medicine, University of Basel, Klingelbergstrasse 61, 4056 Basel, Switzerland; c University of University of Basel, Switzerland; d Armauer Hansen Research Institute, Jimma Road, PO Box 1005, Addis Ababa, Ethiopia; e Seegarten Clinic, Seestrasse 155A, 8802 Kilchberg ZH, Switzerland

**Keywords:** Myalgic encephalomyelitis/chronic fatigue syndrome, Switzerland, Demography, Symptoms, Co-morbidity

## Abstract

Myalgic Encephalomyelitis/Chronic Fatigue Syndrome (ME/CFS) is a multi-factorial systemic chronic debilitating disease of poorly understood etiology and limited systematic evidence. The questionnaire and interview-based survey included 169 ME/CFS patients from the Swiss ME/CFS association. The majority of patients were females (72.2%), single (55.7%) and without children (62.5%). Only one third were working (full/part-time). The mean onset of ME/CFS was 31.6 years of age with 15% of patients being symptomatic before their 18th birthday. In this cohort, patients had documented ME/CFS for a mean 13.7 years, whereby half (50.3%) stated their condition was progressively worsening. Triggering events and times of disease onset were recalled by 90% of the participants. An infectious disease was associated with a singular or part of multiple events by 72.9% and 80.6%, respectively. Prior to disease onset, a third of the patients reported respiratory infections; followed by gastro-intestinal infections (15.4%) and tick-borne diseases (16.2%). Viral infections were recalled by 77.8% of the respondents, with Epstein Barr Virus being the most commonly reported agent. Patients self-reported an average number of 13 different symptoms, all described specific triggers of symptoms exacerbation and 82.2% suffered from co-morbidities. This study collated clinically relevant information on ME/CFS patients in Switzerland, highlighting the extent of disease severity, the associated factors negatively affecting daily life activities and work status as well as potential socio-economic impact.

## 1 Introduction

Myalgic Encephalomyelitis/Chronic Fatigue Syndrome (ME/CFS) is a chronic debilitating systemic neuro-immunological disease. The exact etiology remains unknown, but thought to be multi-factorial [[1], [2], [3], [4], [5], [6], [7], [8]]. Emerging evidence points towards immune and inflammatory pathologies, chronic neuro-inflammation, cell receptor anomalies, decreased metabolism and mitochondrial dysfunction in ME/CFS patients [[9], [10], [11], [12], [13], [14], [15], [16]]. The lack of reliable biological markers hampers definitive diagnosis, which is currently still made after exclusion of a set of diseases and clinically-based following existing case-definitions [[17], [18], [19]].

Reported symptoms relate to immune-autonomic-neurological and endocrinological impairments, and most commonly include profound fatigue, post-exertional malaise (PEM), orthostatic intolerance, unrefreshing sleep and cognitive impairment [2,20]. Symptoms last longer than 6 months, worsen with physical and/or mental exercise and have prolonged recovery phases [[21], [22], [23], [24], [25], [26]].

The globally reported ME/CFS prevalence ranges between 0.2% and 2.8% [[27], [28], [29], [30], [31], [32]]. A recent systematic review conducted by Estevez-Lopez (2020) showed that ME/CFS prevalence ranged between 0.1% and 2.2% in Europe [33].

Despite the severe symptom burden, long duration and associated disabilities caused by the disease, Switzerland remains one of the few European countries for which, to our knowledge, highly limited systematic scientific data on ME/CFS patients exists and its disease prevalence remains unknown. The objective of this study was to collate for the first time evidence on issues relevant to ME/CFS in a selected population of ME/CFS sufferers (e.g. demographic indicators; disease information), who were both Swiss resident and members of the Swiss ME/CFS association, in order to promote awareness and recognition of ME-CFS in Switzerland.

## 2 Material and methods

### 2.1 Study design and participants

This cross-sectional study was carried out between June and September 2021 in Switzerland by a mixed gender research team with practical and research medical and epidemiology background (MD; Prof; PhD). There is currently no disease register for ME/CFS patients in Switzerland. Participants were consenting ME/CFS patients aged 18 and older and recruited through the largest Swiss ME/CFS association, which counted over 320 members at the start of the study. The sampling approach was purposive; hence, no sample size calculation nor randomization procedures were required.

All members of the national Swiss ME/CFS Association were informed about the study (via mailing lists, newsletter, homepage and direct information during Association meetings). In total 207 patients declared an interest to participate in the survey. Detailed information about the study was sent individually by post, including the questionnaire and a consent form. Written informed consent was requested from all participants prior to participating in the study. Completed consent forms and questionnaires were sent back using pre-stamped envelopes.

### 2.2 Inclusion/exclusion criteria

Patients who received an ME/CFS diagnosis from health personnel were included in the study. In addition, the study team conducted a quality control in order to ensure reported symptoms did fit the criteria of any of the three case-definitions (CCC, ICM or IOM). Patients not having been diagnosed for ME/CFS by physicians and/or not fulfilling either of the three case definitions for ME/CFS were not included into this study.

### 2.3 Ethical clearance

This research received approval from the Ethics Committee of Northwestern and Central Switzerland (EKNZ, Switzerland), with copy to all other relevant Ethics Committees in Switzerland (Basec nr. 2021-01098; July 2021).

### 2.4 Questionnaire survey

A paper questionnaire survey was self-administered by participants because of the on-going COVID-19 pandemic. Participants could fill the questionnaire at their pace within a two months deadline, which also allowed severely ill ME/CFS patients to participate.

One coder was responsible to code each questionnaire using a unique numerical identification number. The questionnaire was prepared in German and French and pre-tested with four ME/CFS patients who did not participate in the survey, to ensure questions were well designed and well understood. The questionnaire included 66 questions, with closed and open-ended questions pertaining to the following topics: general demography, disease history, therapies, socio-economic impact of the disease, coping mechanism and the impact of the COVID pandemic. Only a section of the questionnaire is presented in this manuscript. In addition, participants were invited in an open-section to share their own insights, in order to gain additional information about their disease that would otherwise not be covered by the structured nature of the questionnaire.

### 2.5 Data management and statistical analysis

Questionnaire data were entered into Microsoft Access and analyzed using STATA software version 16.1 (StataCorp LLC, USA). Descriptive statistical analysis was used to describe the study population and group comparisons. Additional qualitative data collected in the questionnaire in form of open-ended questions were entered into Microsoft Excel and analyzed descriptively by content and relevant participants illustrative quotes presented to support/complement the quantitative questionnaire data analysis. Illustrative quotes are labeled by gender and age of the participant. Qualitative data from the open-section where participants could express in written accounts, experiences or opinions was analyzed thematically.

## 3 Results

In total, 172 participants out of the 207 who wanted to participate in the survey consented and returned completed questionnaires (response rate of 83%). Three participants were excluded from the final analysis (self-reported disease with symptoms not fulfilling any of the ME/CFS case-definitions), resulting in a total sample size of 169 participants.

### 3.1 Demography

The study represented 20 different cantons, with the highest participant numbers from Zurich (N = 48; 28.4%), Bern (N = 32; 18.9%), Aargau (N = 17; 10%) and Lucerne (N = 10; 5.9%). There were no participants from the cantons Uri, Obwalden, Appenzell Innerrhoden, Glarus, Neuchatel and Ticino.

The majority were women (N = 122; 72.2%). The great majority (N = 148; 87.6%) were in the working age (25–64 year). Detailed ME/CFS cohort demographic data is summarized in Table 1.

**Table 1:** Demography of Swiss ME/CFS participants.

Category	Sub-category	Number (%)
Sex (N = 169)	Female	122 (72.2)
	Male	47 (27.8)
Age in years (N = 169)	13–18	4 (2.4)
	19–24	8 (4.7)
	25–44	59 (34.9)
	45–64	89 (52.7)
	≥65	9 (5.3)
Marital status (N = 169)	Single	68 (40.2)
	Married	54 (32.0)
	Partnership	21 (12.4)
	Divorced	23 (13.6)
	Widowed	3 (1.8)
Having children (N = 168)	No	105 (62.5)
	Yes	63 (37.5)
Education (N = 169)	Compulsory school	13 (7.7)
	Matura or vocational training	82 (48.5)
	University Bachelor	22 (13.0)
	Higher education (MSc/PhD/Prof)	35 (20.7)
	Other	17 (10.1)
Work status (N = 168)	Working	51 (30.3)
	Sick leave	49 (29.2)
	Not working (on disability insurance)	38 (22.6)
	Retired	13 (7.7)
	Unemployed	7 (4.2)
	Other	10 (6.0)
Learned profession categorized (N = 160)	Business and administration	40 (25)
	Academic professions	36 (22.5)
	Medical professions	28 (17.5)
	Teaching	23 (14.4)
	Tradesmen	13 (8.1)
	Technicians	12 (7.5)
	Service professions	5 (3.1)
	Agriculture and gardening	3 (1.9)

### 3.2 Disease history

Mean age of disease onset overall was 31.6 years (95%CI: 29.7–33.4), ranging from 7 to 69 years. In the majority (N = 117; 69.6%) of respondents, ME/CFS started during the most productive work years (25–64 years). In 25 patients (14.7%), the disease started before the 18th birthday (Table 2). At the time of the study, patients had been living with ME/CFS with a mean 13.7 years (SE: 0.73; 95% CI: 12.2–15.1). Over half of the patients (N = 94; 56.3%) had ME/CFS for over 10 years.

**Table 2:** Age at disease onset and number of years living with ME/CFS.

Category	Sub-category	Number (%)
Age at onset (years)	6–12	6 (3.6)
	13–18	19 (11.3)
	19–24	25 (14.9)
	25–44	97 (57.7)
	45–64	20 (11.9)
	≥65	1 (0.6)
Number of years living with ME/CFS	1–3	23 (13.8)
	4–10	50 (29.9)
	11–20	57 (34.1)
	21–30	24 (14.4)
	>30	13 (7.8)

### 3.3 Perceived associative events of ME/CFS onset

Hundred-fifty-two participants (90%) could recall an associative event with the start of their disease (Table 3). The majority of these patients (N = 117; 69.2%) recalled an infectious disease (i.e. febrile illness) as the most prevalent association prior the onset of ME/CFS.

**Table 3:** Recalled event(s) associated with the onset of ME/CFS (N = 152).

Event	Number (%)
Infectious diseases	117 (77)
Stress	63 (41.1)
Emotional trauma	29 (19.1)
Surgical procedure	23 (15.1)
Physical trauma	15 (9.9)
A stay abroad (work; holiday)	14 (9.2)
Depression	13 (8.5)
Others	28 (18.4)

Among the 117 patients stating infectious diseases as an onset for ME/CFS, 69 (59%) mentioned Epstein-Barr-virus (EBV). Of these, 46 had a confirmed clinical disease, whereas 23 had a sub-clinical infection (antibodies for EBV found during routine blood test; ME/CFS examination). Viral diseases were mentioned by the majority of participants (N = 91; 77.8%), followed by respiratory infections (N = 39; 33.3%), Gastro-intestinal infections including hepatitis (N = 18; 15.4%), tick-borne diseases (N = 19; 16.2%) and miscellaneous infections (N = 20; 17.1%). Table 4 shows the detailed diseases reported by the patients.

**Table 4:** Diseases reported by patients perceived to be associated with the onset of their ME/CFS (N = 117).

Infectious disease	Total	Categories	Number	Remarks
Viral diseases	91	Herpesviridiae	84	Clinical EBV (N = 46); subclinical EBV (N = 23); Herpes spp (N = 12); Cytomegaly Virus (N = 3)
		Childhood diseases	6	Complicated chickenpox (N = 2); Measle (N = 1); Mumps (N = 1); Rubella (N = 1); Scarlett fever (N = 1)
		Unspecific	1	
Respiratory infections	39	Common colds and flu	28	Severe; protracted
		Pneumonia	9	
		Tuberculosis	1	
		SARS virus	1	
Gastro-intestinal infections	19	Pathogens and parasites	10	Helminthiasis, Schistosomiasis; Giardia, Campylobacter, Shigella, Helicobacter (all with severe diarrhea and often after trips abroad)
		Gastro-intestinal “flu”	5	Severe; protected
		Hepatitis	4	Hepatitis A (N = 3), EBV complication (N = 1)
Tick-borne diseases (TBDs)	19	Borreliosis	14	
		Tick-borne viral encephalitis	2	
		Other TBDs	3	Ehrlichiosis (N = 1); Bartonella (N = 1); Rickettsia (N = 1)
Sexually Transmitted Diseases (STDs)	2	Chlamydia	1	
		Papiloma Virus	1	
Miscellaneous	17	Upper respiratory tract infections	10	Ear/sinusitis/tonsillitis (N = 7); Purulent throat infections with abscess (N = 3)
		Unspecific bacterial infections	5	
		Mycoplasma	2	

Fifty-nine patients (38.8%) recalled a single event as the start of ME/CFS, whereas a combination of events happening around the same time, was stated by 93 participants (61.2%).

Among the single associative events, the majority reported infectious diseases (N = 43; 72.9%). Less often recalled were: an episode with perception of “extreme stress” (N = 5; 8.5%), a surgical procedure (N = 2; 3.4%), emotional trauma (N = 1; 1.7%) and a visit abroad (N = 1; 1.7%). Seven (11.9%) patients stated “other” causes as a single event (e.g. vaccination; cancer treatment). Depression and physical trauma were never stated as a single event.

Two, three and more than three coinciding events were reported by 56 (36.8%), 23 (15.1%) and 14 (9.2%) of the participants, respectively. Among this sub-group of multiple associative events, infectious diseases were reported by 75 (80.6%) of the patients. Other events (pooled into the category “other”) included three cancer (mamma carcinoma, intestinal cancer); 13 vaccinations; two drug induced (mephloquine, long term fluorchinolones); one acute urinary retention; one mitochondriopathy; three laboratory incidents (exposure to mercury and nitrous oxide); one surgical heart catheter implant with subsequent complications).

Sixteen patients mentioned vaccines to have been associated with the onset of ME/CFS. It included: six Hepatitis B (inoculation after year 2000); three pox; one tick-borne encephalitis; two influenza; one swine flu; one typhus; one tetanus; one Yellow fever given at the same time as meningococcus). Four patients stated vaccines as the perceived sole event linked to the onset of ME/CFS (three Hepatitis B, one simultaneous Yellow Fever and Meningococcus vaccination).

A trip abroad reported as association with the onset of ME/CFS included destinations to Africa, South-East Asia, Central Asia, South-America, and Northern Australia.

### 3.4 Disease start and disease course over the years

The disease started progressively (within one year) in 63 of 166 participants (38%), whereas 61 (36.7%) and 42 (25.3%) stated the symptoms appeared within one month and above one year, respectively. Eight patients mentioned a rapid initial onset but a slow or very slow worsening of symptoms after sub-sequent trigger events, such as surgical procedures or additional infections.

Half of the patients (N = 85; 50.3%) described a continuous worsening of symptoms over the years (with or without fluctuation). Seventy-eight (54.1%) described the disease as fluctuating with remission and “crash” periods. Improvement of the symptoms over time were described by 35 (20.7%) patients. Once the ME/CFS symptom complex had developed, none of the patients has fully recovered. Over half of the respondents (N = 95; 56.2%) had been bed-bound due to ME/CFS for a longer period at one stage of their life and 44 (26.3%) had to be hospitalized at least once for ME/CFS symptoms. Among the women who were pregnant after the onset of ME/CFS, seven (30.4%) claimed that the pregnancy worsened the ME/CFS symptoms, five (21.7%) said there was no change in disease severity and four (17.4%) stated that ME/CFS symptoms improved during pregnancy. The other eight women could not recall the influence of pregnancy on their ME/CFS.

Two respondents stated that severe muscle weakness led to a fall with subsequent bone fractures. One severe bed-bound ME/CFS patient (22 years old) passed away after the completion of the study.

Three patients observed that their child/children have also developed ME/CFS (mentioned possible association with the disease onset were EBV, Lyme disease, vaccination). One participant mentioned his brother having developed the illness as well.

### 3.5 Reported symptoms of ME/CFS

All respondents reported profound debilitating fatigue. Table 5 summarizes the reported symptoms divided into broad categories as a closed question asked to the participants. The other top 5 five symptoms were pain (89.3%) (including myalgic, joint, head, eyes, thorax etc.), cognitive impairments (88.2%), Post-Exertional malaise (PEM) (85.2%) and noise sensitivity (82.2%). Table 6 shows detailed self-reported symptoms as perceived by patients to be their main symptoms besides fatigue and PEM (answering to an open question). Additionally, respondents listed the top five most disabling symptoms as being fatigue (N = 96; 56.8%), followed by PEM (N = 38; 22.5%), cognitive impairment (N = 24; 14.2%), pains (N = 10; 5.9%) and muscle weakness (N = 9; 5.3%). The number of clearly discernible symptoms experienced by the patients ranged between three and 22, with a mean of 13 symptoms (95%CI:12.4–14.7).

**Table 5:** Symptom classification (N = 169).

Symptom category	Number (%)
Fatigue	169 (100)
Pains (muscle, joints, including headaches/migranes)	151 (89.3)
Cognitive impairments	149 (88.2)
Post-Exertional malaise (PEM)	144 (85.2)
Noise sensitivity	139 (82.2)
Sleep disturbance	130 (76.9)
Muscle weakness	127 (75.1)
Gastro-intestinal symptoms (e.g. vomiting, nausea, diarrhea, constipation, bloating)	110 (65.1)
Light sensitivity	106 (62.7)
Neurological symptoms	100 (59.2)
Neurological sensory symptoms	94 (55.6)
Alcohol intolerance	94 (55.6)
Feeling hot	91 (53.8)
Heart symptoms	88 (52.1)
Feeling cold	86 (50.9)
Respiratory symptoms	80 (47.3)
Sore throat	77 (45.6)
Odor intolerance	67 (39.6)
Drug intolerance	63 (37.3)
Enlarged/painful lymph nodes	59 (34.9)
Sub-febrile/fever bouts	49 (29.2)
Other symptoms	30 (17.7)

**Table 6:** Self-reported main symptoms besides fatigue and PEM, experienced by patients (N = 154).

Category	Description	Reported number
Sleep disturbance	Insomnia/sleep rhythm disturbances	24
	Unrefreshing sleep	16
	Increased need for sleep	12
Neurocognitiv impairment	Brain fog	61
	Concentration impairment	37
	Difficulty finding words	5
	Slurry speech	5
	Unspecified	2
Neuroendocrine manifestation	Recurrent sub-febrile/febrile episodes	11
	Poor thermoregulation	7
	Anxiety	7
	Feeling always cold	4
	Heat/cold intolerance	2
	Emotional sensibility	2
	Lack of appetite	1
	Sweating	1
Neurological Symptoms	Motor disturbance - Muscle weakness (unable to stand for long, difficulty walking, need of a walking stick of wheelchair, it feels like a “paresis”	86 78
	- Muscle cramps	2
	- Poorly coordinated movements	6
	Sensitivity to light/noise	29
	Tinnitus	8
	Visual impairments (blurry vision; inability to focus)	6
	Neuropathy (paresthesia)	4
	Seizure-like cramps	2
	Extremety numbness	1
	Tremors (hand, head)	1
	Feeling like having had a stroke on the left side	1
	Phantom smells (e.g. aceton)	1
Pain	Muscle/joint	98
	Headache/migranes/increased pressure behind the eyes or ears	39
	Thorax pains	1
Immunological manifestation	Feeling sick/flu-ish	18
	Regular bouts of temperature or fever	7
	Food allergies/intolerances	7
	Recurrent sore throat	4
	Recurrent swollen lymph nodes	4
	Recurrent sinusitis	3
	Drug intolerance	2
	Alcohol intolerance	1
	Mouth lesions/ulcers	1
	Susceptibility to infections	1
	Recurrent ear-nose-throat infections	1
Gastrointestinal manifestation	Diarrhea, constipation, bloating, vomiting	37
	Nausea	13
Autonomic manifestation	Dizzyness	26
	Orthostatic intolerance/POTS	10
	Palpitations/tachycardia	3
	Hypotension	2
	Bladder irritability	2
	Syncope	1
	Autonomous symptoms	1
	Eye irritation	1
Metabolic/Endocrine	Long menstrual cycle	1
	Metabolic HPU	1
Respiratory system	Short of breath, painful breathing	13

All participants (N = 169) reported “triggers” that started or aggravated their symptoms (Table 7). The top four triggers were physical activity (88.7%) followed by general stress (82.8%), lack of sleep (79.3%) and mental activities (70.4%).

**Table 7:** List of triggers described to aggravate symptoms (N = 169).

Trigger type	Number (%)
Physical activity	150 (88.7)
Stress	140 (82.8)
Lack of sleep	134 (79.3)
Mental activity	119 (70.4)
Noise	99 (58.6)
Heat	68 (40.2)
Cold	49 (29.0)
Diet	42 (24.8)
Other*	25 (14.8)

Note: (*) Among the category “other”, following triggers were mentioned: vaccination, infectious disease; menstruation, crowds of people, travels, medication, bright light, high altitude, alcohol consumption.

After a trigger, symptoms progressively worsened most commonly in a delayed manner. The majority of the respondents said that the peak of the “crash” usually happened 24 h later (N = 122; 72.6%). Whereas, 46 (27.4%) observed crashes after 48 h and 14 (8.3%) after several days. Depending on the severity of the trigger intensity, some symptoms were also said to worsen within hours of the trigger (N = 96; 57.1%). All stated that severity and length of the trigger was an important determinant of resulting crashes.

Similarly, recovery time depended on the severity of a crash. The majority stated that recovery lasted around one week after an exacerbation (N = 95; 56.2%). Seventy-seven (45.6%) said that it took one to two days with mild triggers, whereas 51 (30.2%) said it would take 2–4 weeks and 25 claimed it would take over one month (N = 25; 14.8).

### 3.6 Self-reported Co-morbidities

The majority of patients (N = 139; 82.2%) suffered from co-morbidities. Of these 137 patients named the type of co-morbidity (Table 8). The most commonly reported co-morbidity was allergies (N = 56; 40.3%). Among other co-morbidities, the following were named: hypermobility syndrome, Scheuermann's disease, Marfan Syndrome, Ehlers Danlos Syndrome, Sjogren's syndrome, asthma, mast cell activation syndrome, eosinophilic syndrome, multiple chemical sensitivity syndrome and Adult attention deficit/hyperactivity disorder (ADHS).

**Table 8:** List of co-morbidities in the study population (N = 137).

Co-morbidity	Number (%)
Allergies	56 (40.3)
Irritable bowel syndrome (IBS)	49 (35.2)
Migraine	42 (30.2)
Clinical depression	32 (23.0)
Fibromyalgia (FM)	31 (22.3)
Thyroid diseases	23 (16.5)
Borreliosis	9 (6.5)
Diabetes mellitus (DM)	6 (4.3)
Multiple sclerosis	1 (0.7)
Other	48 (34.5)

### 3.7 Activity reduction as per pre-disease

All participants reported that ME/CFS led to a reduction of activity level, either at work or at home or both. The great majority (N = 162; 95.8%) stated a decrease in activity level of 50% and above as compared to the pre-disease period. Among them, over half (N = 87; 53.7%) were severely affected (mostly house-bound to bed-bound).

After exclusion of 13 people (for this sub-analysis) who were retired at the time of the study, the majority of respondents were not working at all (N = 92; 59.7%). Seven (4.5%) had a full-time job and 55 (35.7%) had a part-time employment. With the exception of one respondent (0.7%), all stated that ME/CFS was the reason or partial reason for decreased workload.

All major life activities were affected by ME/CFS. The majority of respondents stated that it affected mostly their social/friendship life (N = 166; 98.2%) and hobbies/free time (N = 166; 98.2%). This was followed by impact on carrying on household chores (N = 156; 92.3%), on their work (N = 149; 88.1%), family life (N = 147; 87.0%), marital life (N = 131; 77.5%) and on other aspects (N = 31). Ability to perform and carrying on with normal daily life activities in terms of time and intensity as per pre-disease times was mentioned by 5/166 (3%) people for household chores, one out of 160 (0.6%) for social/friendship life and none for free-time activities and hobbies. We attempted to assess the reduction of these activities but due to large variations depending on the remission and crash phases, it was impossible to quantify accurately. Patients often described how they made daily or weekly plans for activities, with each day dedicated to one activity only. A 31 year-old female described: *“Depending on the days, I can do one to 3 h of activities. So each day I can only chose one activity (e.g. showering, go shopping, cleaning the house, social life etc)”.* While another participant (female, 38 years) mentioned: *“I can only do 15 min per activity, then I have to rest”.* Others described how they had to make choices, if they chose work, then they could not do any other activity that day; if they chose another activity, for instance a hobby or socializing, then they could not work. This was reflected by the statement of a 41 year-old female participant who said: “*“I have 30–40% energy I can spend per day; I have to choose what to do with it; if I work, I cannot do other things; If I do social activities, then I cannot work”.*

## 4 Discussion

There is a substantial amount of scientific literature on ME/CFS in the public domain, and the disease entity has been recognized as an immune-neurological chronic and debilitating disease for over half a century. Nevertheless, ME/CFS has remained in the shadows of health systems due to lack of a clear-cut diagnostic approach, paucity of official recognition in some countries, absence of defined biomarkers, and limited societal and professional disease knowledge.

To our knowledge, this is the first systematic study in a defined ME/CFS study population in Switzerland. Demographic results were similar to the ones previously reported by other countries [34]. Although all sex and age groups were affected, females represented the great majority of patients (72%), patients were mostly single (55.7%) and had no children (62.5%). Comparing these figures to the general Swiss population (76% married/partnership; 34% with no children) these findings are striking and raise the question to what extend ME/CFS does affects relationships and the ability to raise a family in Switzerland [35]. There appeared no selection for patients according to their educational background or work position, although a noticeably high number of participants were observed in the medical profession – of which half were nurses (Table 1, data not shown), or in the teaching profession. Approximately one fifth of the Swiss ME/CFS patients had a higher education degree (MSc, PhD), which was higher than observed in the Danish ME/CFS population [34].

The exact etiologies for ME/CFS remain unknown and debated, but are clearly multifactorial. One major reason for this is that the associations remain descriptive, and functional pathophysiological links are lacking – this is reflected by the diagnosis which “*per exclusionem*” and the absence of defined biomarkers, which would provide this disease with more evidence and lead to more acceptance by medical staff. Popular hypotheses for triggers involve infectious diseases, toxins, traumas, or genetic predisposition – but clinical studies proving or interlinking these – or interventions affecting these – are not available in the peer-reviewed literature [2]. Inheritage requires more attention, as four patients reported close relatives also suffering from the illness, supporting a possible underlying genetic predisposition as suggested by previous studies [[36], [37], [38]]. Mitochondrial dysfunctions with subsequent energy metabolism impairment have repeatedly been described in ME/CFS patients-at least in a sub-group of patients [[39], [40], [41]]. Mitochondrial DNA mutations are maternally inherited disorders [42]. Venter et al. (2019) observed a mitochondrial genetic difference between ME/CFS patients and controls, supporting the possibility of genetic linkage in a sub-group of patients [43]. The exact role of these genetic variants are still unclear, but it has been proposed that they could modulate susceptibility to disease or the course of a disease [43].

The moment of onset appears to be memorable, as over 90% of participants could clearly describe when and how the disease started. This is consistent with the study by Chu et al. (2019) who also reported that 85% and 88% of the patients remembered a specific time and reason for the disease onset, respectively [38]. The likelihood of this being an infectious disease or an intense period of perceived “stress” is high, and underlined by patients’ reports in our study, with 69% and 41% reporting these associations. Similarly, in the US, 64% and 39% respondents reported an infectious illness and stress as disease onset [38,44]. Although more than a third could pinpoint the onset to a single event, almost two thirds described a combination of events at the time of disease onset. This complicates the fact, whether an event should be seen as primary cause or rather a precipitating or facilitating factor. In both single and multi-event cases, infectious diseases were the most relevant association with ME/CFS with approximately three quarters remembering this. It must be noted that physical trauma and depressive state were never mentioned as a single event.

Patients then reported subsequent events leading to progression and marked worsening of the symptoms. Again, stress, infectious diseases and surgery figured here, but also vaccination (most commonly Hepatitis B) was associated with perceived gradual worsening of symptoms. There is an increasing body of research looking into an association between vaccines and ME/CFS but no causal explanation has been reported [[45], [46], [47], [48]]. Aluminum adjuvants were debated, as the non-biodegradable aluminum-salt crystals serve to strengthen inflammatory responses to improve vaccine immunogenicity [49,50]. It appears justified to hypothesize that infection- or vaccination-induced inflammation could trigger or exacerbate an adverse development of ME/CFS - especially as in some patients with underlying immune impairments, the precipitating or contributing effect appears more pronounced. Worldwide, ME/CFS official guidelines recommend-similarly to immunocompromised patients-not using live vaccines in ME/CFS patients [51,52].

Interestingly, viral infections were mentioned by almost 80% of respondents with clinical and subclinical Epstein Barr infections most frequently reported. EBV of the Herpesvirus family is one of the most common human viruses. We could not compare the results with the EBV prevalence in the general Swiss population for which data is lacking [53]. A recent Bulgarian study, however, showed that EBV infections were statistically significantly more frequent in ME/CFS patients than in control groups [54]. The role of EBV in ME/CFS has been often been debated. Recent research suggest that EBV may be an important risk factor for the development of ME/CFS – at least in a sub-group of patients, linked with immune dysfunctions and/or genetic predisposition, and could contribute to the observed neuro-immunological impairments seen in ME/CFS patients [[54], [55], [56], [57]]. A review by Rasa et al. (2021) showed that viral diseases such as human herpesviruses, enteroviruses and huan parvovirus B19 were associated with ME/CFS onset and likely caused the irreversible immunological impairments observed, rather than causing chronic or latent viral infections [58]. Several other agents have been associated with ME/CFS in the literature (e.g. Borrelia, Q-fever, Brucellosis, MCV). To date, no single agent has been identified in all ME/CFS patients, again suggestive that the underlying immune response or inflammation require more attention. The infectious diseases associated with the onset or progression of ME/CFS could represent etiology and/or risk factor. Nevertheless, a third of the patients reported respiratory diseases – usually severe or protracted (flu, colds, pneumonia) – and the recent COVID-19 pandemic caused by SARS-Cov-2 virus also led to chronic “long” COVID-19 cases with similar symptomology to ME/CFS. The similarity and possible relationship have been flagged by institutions such as WHO, CDC, and the NIH. Researchers estimate that COVID-19 infections could be a trigger for the onset of ME/CFS [1], while others rather point towards a reactivation of EBV by the COVID-19 virus [59].

Concomitant diseases and syndromes are not uncommon in ME/CFS patients and can complicate the diagnostic, management and treatment. The majority of our respondents (82.2%) suffered from at least one co-morbidity. The most important ones were allergies and irritable bowel syndrome (IBS), migraine, depression, fibromyalgia and thyroid diseases. This is in line with a Spanish cohort study that found that over 80% of their subjects had co-morbidities [60]. Similarly, Bateman et al. (2014) reported 84% of their ME/CFS respondents to be suffering from co-morbidities – such as fibromyalgia, depression, anxiety and hypothyroidism [61].

The mean onset of the disease at 31.6 years of age is in line with data from other countries [19,62,63]. Around 15% of the patients were younger than 18 years, meaning that they will enter adulthood and their productive work years with this chronic disease, and patients often suffer from ME/CFS for the rest of their lives [64]. Our study showed that at the time of the survey, patients were in average already ill for 13.7 years on average, with over half of them (56.3%) having had ME/CFS for over 10 years. Moreover, none had fully recovered and half of the cohort stated that the disease had been getting worse over the years. These results support the urgent need for fast diagnosis and close follow-up of patients, so that overall severities, progression rates and co-morbidities can be reduced to the highest level. A pre-requisite is however, that physicians know and recognize the disease. A review among European countries showed that up to a half of the GPs did not recognize ME/CFS as a medical entity [65]. The European Network on ME/CFS (EUROMENE) has recently produced an expert consensus and recommendations regarding diagnosis and better care and service provision for ME/CFS patients [26].

In light of the reported severity of symptoms, the triggers that worsen the disease course and slow recovery, patients would benefit from the provision of adequate life and work environments that would minimize triggers and support faster recovery from exacerbations. A pre-requisite being better awareness, understanding and acceptance of the disease by the society.

Importantly, this study showed that ME/CFS patients in Switzerland struggle not only with the clinical symptoms, but also consequences of the accompanying disabilities; mental health issues remain under appreciated and require adapted coping strategies – these are fortified further by impaired social life and financial insecurity.

The study strengths were the high response rate of participants thanks to the possibility of questionnaire self-administration, which reflected how patients felt important to be heard and be more visible. The opportunity to give a voice to patients through open-ended questions or sections that could be used freely allowed collecting valuable qualitative information that is often not captured by structured questionnaires. The wide age range of participants as well as the inclusion of high number of house and bed-bound patients contributed to the strength of this study. The limitations were the ones inherent to the self-administered questionnaire and self-reporting of medical information approach, as well as possible recall bias. Although diagnosed by medical professionals, we do not know which case definition the physicians used. Furthermore, we assume an under-representation within the ME/CFS association of patients from French and Italian speaking cantons (German as main communication language), as well as patients suffering from milder forms of ME/CFS. Therefore, a nation-wide cohort study is warranted to corroborate our preliminary findings.

## 5 Conclusion

This study revealed in participating Swiss ME/CFS patients multiple shared common disease features, and highlighted the likelihood of existing sub-groups. The overall burden of the disease was very high (main/accessory symptoms, co-morbidities, socio-economic implications), demonstrating the urgent need for improvement of overall knowledge and acceptance of the disease in healthcare, social care and society as a whole. Inter-disciplinary approaches to improve early diagnosis and long-term care for patients, further research and clinical applications will be mandatory to ensure proper care of the ME/CFS afflicted in Switzerland.

## Author contribution statement

Rea Tschopp: Conceived and designed the experiments; Performed the experiments; Analyzed and interpreted the data; Contributed reagents, materials, analysis tools or data; Wrote the paper.

Rahel S König; Protazy Rejmer; Daniel H Paris: Analyzed and interpreted the data; Wrote the paper.

## Data availability statement

Data will be made available on request.

## References

[1] Poenaru S., Abdallah S.J., Corrales-Medina V., Cowan J. (2021). COVID-19 and post-infectious myalgic encephalomyelitis/chronic fatigue syndrome: a narrative review. Ther. Adv. Infect. Dis..

[2] Centers for Disease Control and Prevention (CDC). Myalgic Encephalomyelitis/Chronic Fatigue Syndrome. https://www.cdc.gov/me-cfs/index.html. Accessed 15 January 2022.

[3] Hickie I., Davenport T., Wakefield D., Vollmer-Conna U., Cameron B. (2006). Post-infective and chronic fatigue syndromes precipitated by viral and non-viral pathogens: prospective cohort study. BMJ.

[4] Hampton T. (2006). Researchers find genetic clues to chronic fatigue syndrome. JAMA.

[5] Myhill S., Booth N., Mclaren-Howard J. (2009). Chronic fatigue syndrome and mitochondrial dysfunction. Int. J. Clin. Exp. Med..

[6] Van Elzakker M. (2013). Chronic fatigue syndrome from vagus nerve infection: a psycho-neuro-immunological hypothesis. Med. Hypotheses.

[7] Glassford J.A.G. (2017). The neuroinflammatory etiopathology of myalgic encephalomyelitis/chronic fatigue syndrome (ME/CFS). Front. Physiol..

[8] Rasa S., Nora Krukle Z., Henning N., Eliassen E., Shikova E. (2018). Chronic viral infections in myalgic encephalomyelitis/chronic fatigue syndrome (ME/CFS). J. Transl. Med..

[9] Sweetman E., Noble A., Edgar C., Mackay A., Helliwell A., Vallings R., Ryan M., Tate W. (2019). Current research provides insight into the biological basis and diagnostic potential for myalgic encephalomyelitis/chronic fatigue syndrome (ME/CFS). Diagnostics.

[10] Brenu E.W., Van Driel M.L., Staines D.R., Ashton K.J., Ramos S.B. (2011). Immunological abnormalities as potential biomarkers in chronic fatigue syndrome/myalgic encephalomyelitis. J. Transl. Med..

[11] Montoya J.G., Holmes T.H., Anderson J.N., Maecker H.T., Rosenberg-Hasson Y. (2017). Cytokine signature associated with disease severity in chronic fatigue syndrome patients. Proc. Natl. Acad. Sci. USA.

[12] Mandarano A.H., Maya J., Giloteaux L., Peterson D.L., Maynard M. (2020). Myalgic encephalomyelitis/chronic fatigue syndrome patients exhibit altered T cell metabolism and cytokine associations. J. Clin. Invest..

[13] Wirth K., Scheibenbogen C. (2020). A unifying hypothesis of the pathophysiology of myalgic encephalomyelitis/chronic fatigue syndrome (ME/CFS): recognitions from the finding of autoantibodies against ß2-adrenergic receptors. Autoimmun. Rev..

[14] Marshall-Gradisnik S.M., Smith P., Brenu E.W., Nilius B., Ramos S.B. (2015). Examination of single nucleotide polymorphisms (SNPs) in transient receptor potential (TRP) ion channels in chronic fatigue syndrome patients. Immunol. Immunogenetics Insights.

[15] Nguyen T., Staines D., Nilius B., Smith P., Marshall-Gradisnik S. (2016). Novel identification and characterization of Transient receptor potential melastatin 3 ion channels on Natural Killer cells and B lymphocytes: effects on cell signaling in Chronic fatigue syndrome/Myalgic encephalomyelitis patients. Biol. Res..

[16] Marshall-Gradisnik S., Huth T., Chacko Anu, Johnston S., Smith P. (2016). Natural killer cells and single nucleotide polymorphisms of specific ion channels and receptor genes in Myalgic Encephalomyelitis/chronic fatigue syndrome. Appl. Clin. Genet..

[17] Fukuda K., Straus S.E., Hickie I. (1994). The chronic fatigue syndrome: a comprehensive approach to its definition and study. Ann. Intern. Med..

[18] Carruthers B.M., Van de Sande M.I., De Meirleir K.L., Klimas N.G., Broderick G. (2011). Myalgic encephalomyelitis: international consensus criteria. J. Intern. Med..

[19] Institute of Medicine (IOM) (2015).

[20] Lim E.J., Son C.G. (2020). Review of case definitions for myalgic encephalomyelitis/chronic fatigue syndrome (ME/CFS). J. Transl. Med..

[21] Van Ness J., Stevens S., Bateman L., Stiles T.L., Snell C.R. (2010). Postexertional malaise in women with chronic fatigue syndrome. J. Womens Health.

[22] Lengert N., Drossel B. (2015). In silico analysis of exercise intolerance in myalgic encephalomyelitis/chronic fatigue syndrome. Biophys. Chem..

[23] Twisk F.N. (2014). A definition of recovery in myalgic encephalomyelitis and chronic fatigue syndrome should be based upon objective measures. Qual. Life Res..

[24] Adamowicz J.L., Caikauskaite I., Friedberg F. (2014). Defining recovery in chronic fatigue syndrome: a critical review. Qual. Life Res..

[25] Rowe P., Fontaine K., Lauver M., Jasion S.E., Marden C.L. (2016). Neuromuscular strain increases symptom intensity in chronic fatigue syndrome. PLoS One.

[26] Nacul L., Authier F.J., Scheibenbogen C., Lorusso L., Helland I.B. (2021). Network on myalgic encephalomyelitis/chronic fatigue syndrome (EUROMENE): expert consensus on the diagnosis, service provision, and care of people with ME/CFS in Europe. Medicina.

[27] Kim C.H., Shin H.C., Won C.W. (2005). Prevalence of chronic fatigue and chronic fatigue syndrome in Korea: community-based primary care study. J. Kor. Med. Sci..

[28] Nacul L.C., Lacerda E.M., Pheby D., Campion P., Molokhia M al (2011). Prevalence of myalgic encephalomyelitis/chronic fatigue syndrome (ME/CFS) in three regions of England: a repeated cross-sectional study in primary care. BMC Med..

[29] Johnston S., Brenu E.W., Staines D. (2013). The prevalence of chronic fatigue syndrome/myalgic encephalomyelitis: a meta-analysis. Clin. Epidemiol..

[30] Valdez A.R., Hancock E.E., Adebayo S., Kiernicki D.J., Proskauer D. (2019). Estimating prevalence, demographics, and costs of ME/CFS using large scale medical claims data and machine learning. Front. Pediatr..

[31] Lim E.J., Ahn Y.C., Jang E.S., Lee S.W., Lee S.H., Son C.G. (2020). Systematic review and meta‐analysis of the prevalence of chronic fatigue syndrome/myalgic encephalomyelitis (CFS/ME). J. Transl. Med..

[32] Jason L.A., Mirin A.A. (2021). Updating the National Academy of Medicine ME/CFS prevalence and economic impact figures to account for population growth and inflation. Fatigue: Biomed., Health Behav..

[33] Estevez-Lopez F., Mudie K., Wang-Steverding X., Bakken I.J., Ivanovs A. (2020). Systematic review of the epidemiological burden of myalgic encephalomyelitis/chronic fatigue syndrome across Europe: current evidence and EUROMENE research recommendations for epidemiology. J. Clin. Med..

[34] Hvidberg F.M., Brinth L.S., Olesen A.V., Petersen K.D., Ehlers L. (2015). The health-related quality of life for patients with myalgic encephalomyelitis/chronic fatigue syndrome (ME/CFS). PLoS One.

[35] Bundesamt für Statistik (2021). Familien in der Schweiz. Statistischer Bericht 2021; Neuchatel. BSF Nr. 1010-2100 (in German). https://www.bfs.admin.ch/bfs/de/home/statistiken/bevoelkerung/familien.assetdetail.17084546.html.

[36] Buchwald D., Herrell R., Ashton S., Belcourt M., Schmaling K. (2001). A twin study of chronic fatigue. Psychosom. Med..

[37] Albright F., Light K., Light A., Bateman L., Cannon-Albright L.A. (2011). Evidence for a heritable predisposition to chronic fatigue syndrome. BMC Neurol..

[38] Chu L., Valencia I.J., Garvert D.W., Montoya J.G. (2019). Onset patterns and course of myalgic encephalomyelitis/chronic fatigue syndrome. Front. Pediatrics.

[39] Tomas C., Brown A., Strassheim V., Elson J., Newton J. (2017). Cellular bioenergetics is impaired in patients with chronic fatigue syndrome. PLoS One.

[40] Esterhuizen K., van der Westhuizen F.H., Louw R. (2017). Metabolomics of mitochondrial disease. Mitochondrion.

[41] Tomas C., Newton J. (2018). Metabolic abnormalities in chronic fatigue syndrome/myalgic encephalomyelitis: a mini-review. Biochem. Soc. Trans..

[42] Gorman G.S., Elson J.L., Newman J., Payne B., McFarland R. (2015). Perceived fatigue is highly prevalent and debilitating in patients with mitochondrial disease. Neuromuscul. Disord..

[43] Venter M., Tomas C., Pienaar I.S., Strassheim V., Erasmus E. (2019). MtDNA population variation in Myalgic encephalomyelitis/Chronic fatigue syndrome in two populations: a study of mildly deleterious variants. Sci. Rep..

[44] Pi I.G., Sein-Echaluce M.L.G., Aubach L.R., Puig-Gros J.T., Solà J.F. (2016). Stressful events in the onset of chronic fatigue syndrome. Rev. Esp. Salud Publica.

[45] Appel S., Chapman J., Shoenfeld Y. (2007). Infection and vaccination in chronic fatigue syndrome: myth or reality?. Autoimmunity.

[46] Magnus P., Brubakk O., Nyland H., Wold B.H., Gjessing H.K. (2009). Vaccination as teenagers against meningococcal disease and the risk of the chronic fatigue syndrome. Vaccine.

[47] Magnus P., Gunnes N., Tveito K., Bakken I.J., Ghaderi S. (2015). Chronic fatigue syndrome/myalgic encephalomyelitis (CFS/ME) is associated with pandemic influenza infection, but not with an adjuvanted pandemic influenza vaccine. Vaccine.

[48] Principi N., Esposito S. (2018). Aluminum in vaccines: does it create a safety problem?. Vaccine.

[49] Exley C., Swarbrick L., Gherardi R.K., Authier F.J. (2009). A role for the body burden of aluminium in vaccine-associated macrophagic myofasciitis and chronic fatigue syndrome. Med. Hypotheses.

[50] Gherardi R.K., Crépeaux G., Authier F.J. (2019). Myalgia and chronic fatigue syndrome following immunization: macrophagic myofasciitis and animal studies support linkage to aluminum adjuvant persistency and diffusion in the immune system. Autoimmun. Rev..

[51] Carruthers B.M., van de Sande M.I., De Meirleir K.L., Klimas N.G. (2012). Myalgic Encephalomyelitis: Adult & Paediatric: International Consensus Primer for Medical Practitioners. http://www.investinme.org/Documents/Guidelines/Myalgic%20Encephalomyelitis%20International%20Consensus%20Primer%20-2012-11-26.pdf.

[52] Wagner N., Assmus F., Arendt G., Baum E., Baumann U. (2019). Impfen bei Immundefizienz. Bundesgesundheitlbl.

[53] Karrer U., Nadal D. (2014). Epstein-Barr Virus und infektiöse Mononukleose. Swiss Med. Forum.

[54] Shikova E., Reshkova V., Kumanova А., Raleva S., Alexandrova D. (2020). Cytomegalovirus, Epstein-Barr virus, and human herpesvirus-6 infections in patients with myalgic еncephalomyelitis/chronic fatigue syndrome. J. Med. Virol..

[55] Loebel M., Strohschein K., Giannini C., Koelsch U., Bauer S. (2014). Deficient EBV-specific B- and T-cell response in patients with chronic fatigue syndrome. PLoS One.

[56] Ruiz-Pablos M., Paiva B., Montero-Mateo R., Garcia N., Zabaleta A. (2021). Epstein-barr virus and the origin of myalgic encephalomyelitis or chronic fatigue syndrome. Front. Immunol..

[57] Ariza M.E. (2021). Myalgic encephalomyelitis/chronic fatigue syndrome: the human herpesviruses are back. Biomolecules.

[58] Rasa-Dzelzkaleja S., Krumina A., Capenko S., Nora-Krukle Z., Gravelsina S. (2023). The persistent viral infections in the development and severity of myalgic encephalomyelitis/chronic fatigue syndrome. J. Transl. Med..

[59] Gold J.E., Okyay R.A., Licht W.E., Hurley D.J. (2021). Investigation of long COVID prevalence and its relationship to epstein-barr virus reactivation. Pathogens.

[60] Castro-Marrero J., Faro M., Aliste L., Sáez-Francàs N., Calvo N. (2017). Comorbidity in chronic fatigue syndrome/myalgic encephalomyelitis: a nationwide population-based cohort study. Psychosomatics.

[61] Bateman L., Darakjy S., Klimas N., Peterson D., Levine S.M. (2014). Chronic fatigue syndrome and Co-morbid and consequent conditions: evidence from a multi-site clinical epidemiology study. Fatigue: Biomed. Health Behav..

[62] Bakken I.J., Tveito K., Gunnes N., Ghadori S., Stoltenberg C. (2014). Two age peaks in the incidence of chronic fatigue syndrome/myalgic encephalomyelitis: a population-based registry study from Norway 2008-2012. BMC Med..

[63] Capelli E., Lorusso L., Ghitti M., Venturini L., Cusa C., Ricevuti G. (2015). Chronic fatigue syndrome: features of a population of patients from northern Italy. Internl. J. Immunopath. Pharmacol..

[64] Cairns R., Hotopf M. (2005). A systematic review describing the prognosis of chronic fatigue syndrome. Occup. Med. (Oxf.).

[65] Pheby D.F.H., Araja D., Berkis U., Brenna E., Cullinan J. (2020). Literature review of GP knowledge and understanding of ME/CFS: a report from the socioeconomic working group of the European Network on ME/CFS (EUROMENE). Medicina.

