# Supplementary material for: Myalgic encephalomyelitis/chronic fatigue syndrome (ME/CFS): A preliminary survey among patients in Switzerland
Source: Heliyon. 2023 Apr 20;9(5):e15595. doi: 10.1016/j.heliyon.2023.e15595 (PMC10149204; doi:10.1016/j.heliyon.2023.e15595)
Supplement: Multimedia component 1 [file mmc1.docx]

**ME/CFS Survey**

1. **Demography**

**1**. Code Number**: _________________**

**2**. City: ______________ Canton: _____________

**3**. Sex F
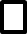
 M

**4**. Age**: ___________________** Year

**5**. Family status (please tick)

|  | Single |  | Partnership |  | Widowed |
| --- | --- | --- | --- | --- | --- |
|  | Married |  | Divorced |  | Other |

**6**. Do you have children?
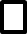
 Yes
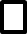
 No

**7**. Profession: 1) learned: __________________ 2) current: _____________________

**8**. What is your highest achieved education? (please tick)

|  | Compulsory school |  | University Bachelor |  | Other |
| --- | --- | --- | --- | --- | --- |
|  | Skilled (vocational)/baccalaureate |  | higher education (MSc, PhD) |  |  |

**9**.What is your current employment status? (please tick)

|  | I work (fulltime/part-time) |  | I am on disability (IV) |  | I am retired |
| --- | --- | --- | --- | --- | --- |
|  | I am unemployed (with benefits) |  | I am on sick leave/long term illness |  | Other |

**10**.Are you a member of the ME/CFS association?
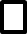
 Yes
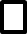
 No

1. **ME/CFS Medical history**

**11**. When did your ME/CFS start? Year: _____________

Or: how old were you? _________________

**12**. Describe briefly your current main symptoms: __________________________________________________________________________

**13**. Do you experience the following symptoms? (only symptoms that you have regularly and over a long period of time) (several answers possible)

|  | PEM* |  | Feeling hot/sweating |
| --- | --- | --- | --- |
|  | Fatigue |  | Heart/circulation problems |
|  | Cognitiv («brain fog, concentration, disorientation, speech etc) |  | Pains (Muscle, joint, headaches, nevralgia) |
|  | Muscle weakness |  | Gastrointestinal symptoms (nausea, vomiting, diarrhea, bloating, abdominal cramps etc.) |
|  | Temperatur/Fever episodes |  | Enlarged or tender lymphnodes |
|  | Neurological symptoms (sight, balance, coordination, falling, stumbling etc.) |  | Sleep disturbance |
|  | Light sensitivity |  | Poor alcohol tolerance |
|  | Noise sensitivity |  | Poor tolerance to odours |
|  | Sensitivity disorder (pins and needles, neuropathies, loss of sensation, painful to the touch, strange feeling on skin etc.) |  | Poor tolerance to medications |
|  | Respiratory disorder |  | Sore throat |
|  | Feeling cold |  | Other |

* PEM (Post-Exertionel Malaise): physical and mental activities lead to a worsening of all symptoms after a latency of several hours to days.

**14**. What is your most disabling symptom? _________________________

**15**. Do you remember any trigger before the disease started? (several answers possible)

|  | No |  | Yes, depression/anxiety |
| --- | --- | --- | --- |
|  | Yes, a physical trauma |  | Yes, I had a surgery |
|  | Yes, an infectious disease (Virus, bacteria, parasite…). Please specify: |  | Yes, I was on a trip/holidays abroad |
|  | Yes, severe stress |  | Other |
|  | Yes, emotional trauma |  |  |

**16**. In which year was your CFS diagnosed? ____________________

(or how old were you?________________)

**17**. How long did it take from your first visit to the doctor to receiving the diagnosis ME/CFS (or til July 2021 if medical diagnosis pending)?

______Month or _____________Year

**18**. Who diagnosed you with ME/CFS? (several answers possible)

|  | Self-Diagnose |  | Specialized doctor |  | Naturopath |
| --- | --- | --- | --- | --- | --- |
|  | GP |  | Another person (Family, friend, colleague) |  | Other |

**19**. Do you remember how the diagnose was made? *(several answers possible)*

|  | Based on assumptions |  | I do not remember |
| --- | --- | --- | --- |
|  | Based on symptoms (and exclusion) |  | Not applicable |
|  | Based on medical/laboratory tests |  |  |

**20**. Did you know ME/CFS before being diagnosed?
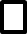
 No
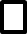
 Yes

**21**. Have you ever been bed-bound for a longer period (weeks-months-years) because of ME/CFS?


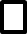
 No
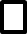
 Yes

(please tick and if possible write the duration)

|  | ______week |  | ______month |  | __________year |
| --- | --- | --- | --- | --- | --- |

**22**. Have you ever been hospitalized because of ME/CFS?


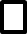
 No
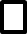
 Yes

**23**. If you are a woman and have children, how did ME/CFS affect your pregnancy(ies)? (*please tick*)

|  | No change |  | Improvement of ME/CFS symptoms |
| --- | --- | --- | --- |
|  | Worsening of ME/CFS symptoms |  | I don’t know |

**24**. How did the disease change over time? (please tick)

|  | Improved |  | Worsened |
| --- | --- | --- | --- |
|  | Remained the same |  | Fluctuates: once better, once worse |

**25**.How did the disease develop? (please tick)

|  | Rapidly (< 1 month) |  | Rather slowly (<1 year) |  | Very slowly (> 1 year) |
| --- | --- | --- | --- | --- | --- |

**26**.Do you suffer from a concomitant disease? *(several answers possible*)

|  | No |  | Allergies |  | Fibromyalgia |
| --- | --- | --- | --- | --- | --- |
|  | Diabetes |  | Multiple Sclerosis (MS) |  | Migrane |
|  | Thyroid disease |  | Irritable Bowel Syndrome (IBS) |  | Depression |
|  | Borreliosis / Lyme |  | Burn-out Syndrome |  | Other |

1. **Impact of ME/CFS on daily life quality and livelihood**

**27**. If you are in the workforce, what is your current work time load?


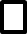
 Full time
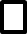
 Part time (%:________)

**28**. If you work part time, is it because of ME/CFS?
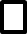
 No
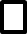
 Yes
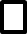
 Partially

**29**. Which aspects of your life are negatively impacted by the disease? (several answers possible)

|  | Friendships/social life |  | Conjugal/partnership life |  | Hobbies/Free time |
| --- | --- | --- | --- | --- | --- |
|  | Household work |  | Work |  | Other |
|  | Family life |  | No negativ effect |  |  |

**30**. Which aspect is your biggest worry in your life/the most affected by ME/CFS (tick only one box)?

|  | Financial security |  | Giving up career |  | Giving up hobbies (sport, garden, travelling etc. |
| --- | --- | --- | --- | --- | --- |
|  | Social relationships |  | Family life |  | Other |

**31**. Do people in your direct life surrounding know about ME/CFS and understand the disease?


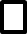
 No
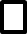
 Yes
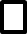
 Sometimes

If rejected, what were the reasons provided? ________________________________________________________________________

________________________________________________________________________

**32**. What is your activity level as compared to before the disease?

|  | The same |  | Medium (around 50% reduction) |  | Very severe (mostly bed-bound, need help) |
| --- | --- | --- | --- | --- | --- |
|  | Lower (reduced activity level) |  | Severe (mostly house-bound) |  |  |

**33**. What percentage of the daily activities (at home or at work) can you in average carry out? (please tick, if possible write down the number of hours for each activity*)*

|  | 0% | < 25% | Hour | < 50% | Hour | < 75% | Hour | 100% | Hour |
| --- | --- | --- | --- | --- | --- | --- | --- | --- | --- |
| Work |  |  |  |  |  |  |  |  |  |
| Household |  |  |  |  |  |  |  |  |  |
| Hobbies |  |  |  |  |  |  |  |  |  |
| Free time |  |  |  |  |  |  |  |  |  |
| Friendships |  |  |  |  |  |  |  |  |  |

**34**.Are there any triggers leading to worsening of symptoms or even initiating a crisis?


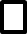
 No
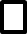
 Yes

*If yes, please tick the corresponding ones, several answers possible*

|  | Cold |  | Noise |  | Mental activities |
| --- | --- | --- | --- | --- | --- |
|  | Heat |  | Physical activities |  | Stress |
|  | Not enough sleep |  | Diet/food related |  | Other |

How long after the triggers, would symptoms start / or get worse?

|  | Immediately |  | After 1 day |  | After 2 days |  | After several days |
| --- | --- | --- | --- | --- | --- | --- | --- |

How long do you need inaverage to recuperate after a crisis?

|  | 1-2 days |  | Around 1 week |  | 2-4 weeks |  | >1 month |
| --- | --- | --- | --- | --- | --- | --- | --- |

**35**. What are the main symptoms that impair carrying out your daily activities? (several answers possible)

|  | Cognitiv/ brain fog |  | Fatigue |  | I don’t know |
| --- | --- | --- | --- | --- | --- |
|  | Pains |  | Muscle weakness |  | Other |

**36**. How do you take care of your household (e.g. shopping, taking care of children, cleaning, cooking)?

|  | I do everything myself |  | I have help from friends |  | Spitex |
| --- | --- | --- | --- | --- | --- |
|  | I have help from my family |  | I hired somebody (eg, cleaner, babysitter, gardener etc.) |  | Other |

1. **Notes/remarks (you can openly write here any further details, information, remarks, notes (it will remain confidential).**
